# Effects of Strength vs. Plyometric Training Programs on Vertical Jumping, Linear Sprint and Change of Direction Speed Performance in Female Soccer Players: A Systematic Review and Meta-Analysis

**DOI:** 10.3390/ijerph18020401

**Published:** 2021-01-06

**Authors:** Elena Pardos-Mainer, Demetrio Lozano, Marcelino Torrontegui-Duarte, Antonio Cartón-Llorente, Alberto Roso-Moliner

**Affiliations:** 1 Health Sciences Faculty, Universidad San Jorge, Autov A23 km 299, Villanueva de Gállego, 50830 Zaragoza, Spain; epardos@usj.es (E.P.-M.); acarton@usj.es (A.C.-L.); aroso@usj.es (A.R.-M.); 2 Department Nursing and Podiatry, University of Malaga, 29016 Málaga, Spain; m.torrontegui@uma.es

**Keywords:** training interventions, fitness assessment, strength and conditioning, football, female

## Abstract

The main purpose of this systematic review and meta-analysis was to compare the effects of strength training (ST) and plyometric training (PT) on vertical jump, linear sprint and change of direction (COD) performance in female soccer players. A systematic search of the PubMed, Web of Science, Google Scholar and SportDiscus databases revealed 12 studies satisfying the inclusion criteria. The inverse-variance random-effects model for meta-analyses was used. Effect sizes (ES) were represented by the standardized mean difference and presented alongside 95% confidence intervals (CI). The magnitude of the main effect was small to moderate (vertical jump (ES 0.53 (95% CI—0.11, 0.95), Z = 2.47 (*p* = 0.01); linear sprint (ES −0.66 (95% CI—2.03, −0.21), Z = 2.20 (*p* = 0.03); COD (ES −0.36 (95% CI—0.68, −0.03), Z = 2.17 (*p* = 0.03)). Subgroup analyses were performed (i.e., ST and PT duration, frequency, session duration and total number of sessions), revealing no significant subgroup differences (*p* = 0.12–0.88). In conclusion, PT provides better benefits than ST to improve vertical jump, linear sprint and COD performance in female soccer players. However, significant limitations in the current literature prevent assured PT and ST prescription recommendations being made.

## 1. Introduction

Women’s soccer has increased in popularity and participation during the last decade [1]. Soccer is considered a contact sport and such impact has had consequences through both a greater skill level and physical demands throughout training and matches [2]. Some of the physical demands for female soccer players during matches have been reported, with total distances covered reaching 10 km, 1.7 km of which was completed at high speed (>18 km·h^−1^) [3,4]. In addition, female players perform between 1350 and 1650 changes of activity, such as passing, tackling, trapping and dribbling [3,4]. Despite its growing popularity, female players are exposed to greater training volumes and competition demands than ever before and, therefore, a better understanding of female players’ physical performance changes is needed to design appropriate training programs.

Female soccer players have been evaluated through a wide variety of physical tests (i.e., Abalakov test, 505 test, linear speed 40 m). These tests can be performed in the laboratory, which is more reliable, and on the soccer field, which is more popular among coaches and physical trainers due to the simplicity and lower cost [2].

Different intervention programs, such as neuromuscular training, plyometric training (PT), strength training (ST) or power training [5,6,7,8], have been performed to improve physical capacities. However, there are discrepancies about which are the best exercises to improve female soccer players’ performance due to the lack of studies.

Plyometrics consists of the rapid stretching of a muscle (eccentric action) immediately followed by a concentric or shortening action of the same muscle and connective tissue [9]. This training method is used to increase strength and explosiveness [10] and it includes a diverse range of bilateral and unilateral jumps, bounds and hops [9]. Regarding female soccer players, PT improves jumping, single and repeated sprinting, changes in direction and kicking power, as well as endurance attributes [11]. Several reviews and meta-analyses related to PT programs have been published in soccer [12,13]. This program constitutes an efficient training solution to improve different power-related skills. However, this evidence has not been clarified in female soccer players, although it has increased the scientific value of PT regarding physical fitness enhancements [12,13]. Hence, more studies for this population are warranted.

Maximal strength is the maximum force or torque that can be exerted by skeletal muscles during movement [14]. An ST program can contribute to improved vertical jump performance, acceleration, leg strength, muscular power, increased joint awareness and overall proprioception [15]. However, intervention studies of ST regarding physical condition in female soccer players are lacking [7]. Despite this, several reviews and meta-analyses related to ST programs have been published in different populations and sports [16,17,18,19]. Nevertheless, the improvement caused by ST raises certain doubts, since the authors do not agree on which doses and exercises are recommended to improve the strength of the lower extremities. In relation to this, research is necessary to provide coaches and practitioners with more information to plan their ST programs.

To our knowledge, there have been no reviews conducted regarding the effects of ST on female soccer players, particularly on physical fitness. Given that PT appears serve as a skill solution to meet the demands of female soccer, an investigation comparing the effects of both programs in female soccer players is warranted. Therefore, the main purpose of this systematic review and meta-analysis was to compare the effects of ST and PT on jump ability, linear sprint and change of direction (COD) performance in female soccer players. A secondary aim was to establish clear guidelines for the prescription of both types of training in female soccer players.

## 2. Materials and Methods

### 2.1. Experimental Approach to the Problem

A systematic review and meta-analysis were conducted following the guidelines of the Cochrane Collaboration [20]. This meta-analytical review was guided by the Preferred Reporting Items for Systematic Reviews and Meta-Analyses (PRISMA) statement [21] and registered in the PROSPERO database with the number CRD42020219998.

### 2.2. Literature Search

**Figure 1:**
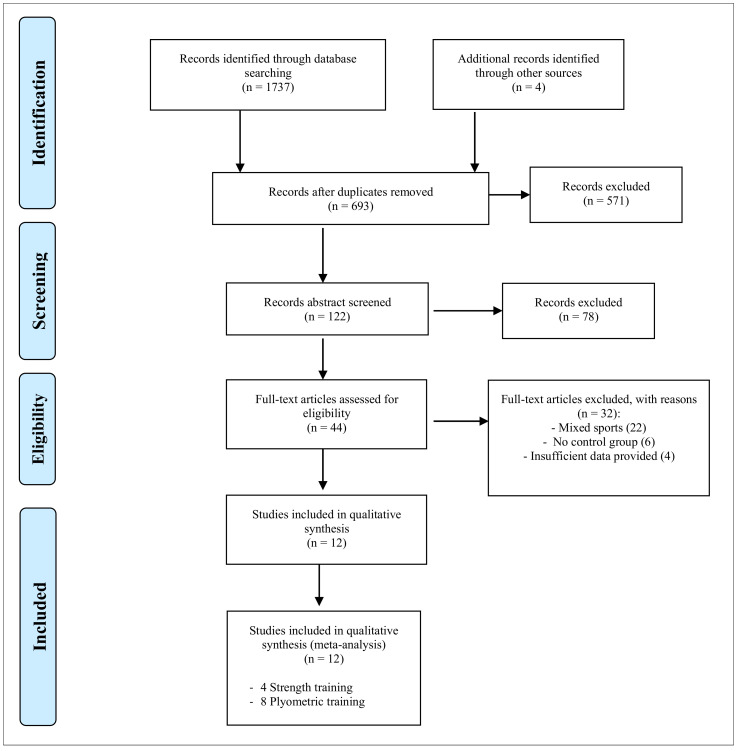
PRISMA flow chart for inclusion and exclusion of studies.

The US National Library of Medicine (PubMed), Web of Science, Google Scholar and SportDiscus electronic databases from inception until 19 October 2020 were searched. Only English and Spanish language articles were considered. Using Boolean logic, we used the following search terms: (“female”) AND (“soccer” OR “football”) AND (“intervention” OR “training”) AND (“strength” OR “plyometric” OR “jump” OR “strength” OR “power” OR “change of direction” OR “side-step” OR “side-cutting” OR “sprint” OR “agility”). In selecting studies for inclusion, a review of all relevant article titles within was conducted before an examination of article abstracts and, then, full published articles. Only peer-reviewed articles were included in the meta-analysis. The search process is outlined in Figure 1. Following the formal systematic searches, additional hand searches were conducted.

### 2.3. Procedures

In selecting studies for inclusion, a review of all relevant article titles was conducted before an examination of article abstracts and then full published articles. Two authors conducted the process independently. Potential discrepancies between the two reviewers about study conditions were resolved by consensus with a third author. Full-text articles excluded, with reasons, were recorded. Data were extracted from gathered articles by two authors independently, using a form created in Microsoft Excel (Microsoft Corporation, Redmond, WA, USA).

The extraction of data from gathered articles was undertaken by two reviewers.

The following criteria determined the eligibility of studies for inclusion in the review: cohorts of healthy female soccer players, with no restriction for age; strength and plyometric interventions must have been at least 2 weeks in duration and must have included a control group (CG) and group mean baseline and follow-up data outcome measures relating to vertical jump, linear sprint and COD performance. The study involved a randomized controlled trial or quasi-experimental design. Based on previous studies, we defined ST as “maximal strength and muscular hypertrophy to improve physical performance” [22] and PT as “lower-body unilateral and bilateral bounds, jumps, and hops that use a pre-stretch or countermovement that incites usage of the stretch-shortening cycle” [23]. A measure of physical fitness was selected based on a logically defensible rationale [24,25], most often some form of countermovement jump (CMJ) without or with arms, linear sprint between 15 and 30 m, V-cut test, 505 test or Illinios Agility test.

### 2.4. Statistical Analyses

Meta-analytical comparisons were carried out in RevMan version 5.3 [26]. Included were 12 studies that comprised 13 individual experimental groups. Means and standard deviations for a measure of post-intervention performance within experimental group (pre- vs. posttest) and between groups (experimental vs. control group) were used to calculate an effect size (ES). Effect sizes were adjusted using Hedges’ small sample size bias correction [27]. The inverse-variance random-effects model for meta-analyses was used because it allocates a proportionate weight to trials based on the size of their individual standard errors [28] and facilitates analysis whilst accounting for heterogeneity across studies [29]. Effect sizes are represented by the standardized mean difference (Hedges’ *g*) and are presented alongside 95% confidence intervals. The calculated ESs were interpreted using the conventions outlined for standardized mean difference by Hopkins et al. [30] (<0.2 = trivial; 0.2–0.6 = small, 0.6–1.2 = moderate, 1.2–2.0 = large, 2.0–4.0 = very large, >4.0 = extremely large).

In cases in which there was more than one intervention group in a given study, the control group was proportionately divided to facilitate comparison across all participants [31].

To gauge the degree of heterogeneity amongst the included studies, the *I*^2^ statistic was referred to. This represents the proportion of effects that are due to heterogeneity as opposed to chance [21]. Low, moderate and high levels of heterogeneity correspond to *I*^2^ values of 25%, 50% and 75%; however, these thresholds are considered tentative [32]. The *χ*^2^ (chi square) statistic determines if any observed differences in results are compatible with chance alone. A low *p* value, or a large *χ*^2^ statistic, relative to its degrees of freedom, provides evidence of heterogeneity of intervention effects beyond those attributed to chance [28].

The Physiotherapy Evidence Database (PEDro) scale was used to assess the risk of bias and methodological quality of eligible studies included in the meta-analysis. This scale evaluates internal study validity on a scale from 0 (high risk of bias) to 10 (low risk of bias) to each methodological item listed in Table 1. A score of ≥6 represents the threshold for studies with a low risk of bias [29].

### 2.5. Analysis of Moderator Variables

To assess the potential effects of moderator variables, subgroup analyses were performed. This method, which was preferred to meta-regression, is based on the documented limitations on the latter method when applied to small datasets with low samples and few predictor variables [33].

Using a random-effects model, potential sources of heterogeneity likely to influence the effects of training were selected a priori. The moderator variables of program duration (weeks), training frequency (sessions per week), total number of training sessions and session duration (minutes) were chosen based on the accepted influence of the FITT (frequency, intensity, type and time) principle on adaptations to exercise [34], as previously demonstrated in meta-analyses performed in female athletes participating in different training interventions [12,35]. Each variable was divided using a median split, except for mean total sessions, in which studies were allocated as groups with more than 16 sessions and groups with less than 16 sessions. Meta-analysis stratification by each of these factors was performed, with a *p* value of <0.05 considered as the threshold for statistical significance.

## 3. Results

### 3.1. Study Selection

A total of 1737 studies were found in the identification phase. After removing duplicates and adding additional records identified through other sources, 693 publications were retained for the article selection process. Title and abstract selection excluded 571 articles. The remaining 44 records were further examined using the specified inclusion/exclusion criteria, and 32 records were subsequently rejected. Finally, 12 studies were included in the systematic review and meta-analysis (Figure 1).

### 3.2. Methodological Quality

The selected studies were submitted to the PEDro methodological quality scale. Two studies obtained a score of 9/10 [36,37], one study obtained 8/10 [38], six obtained 7/10 [5,6,39,40,41], two obtained 5/10 [42,43], and two obtained 4/10 [44,45]. Table 1 displays the complete and detailed PEDro scale score of each study.

### 3.3. Study Characteristics

The characteristics of the participants and ST and PT programming parameters from the 12 studies incorporated in the meta-analysis are indicated in Table 2 and Table 3.

### 3.4. Main Effect

#### 3.4.1. Vertical Jump Performance

**Figure 2:**
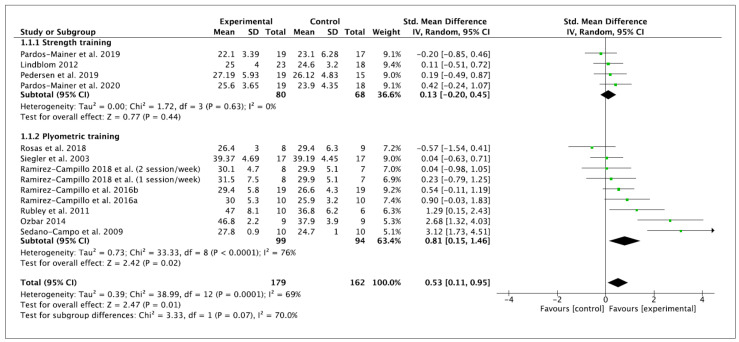
Forest plot of between-mode effect sizes with 95% confidence intervals (CIs) in vertical jump performance (cm). IV: inverse variance method; SD: standard deviation; Std: standardized.

**Figure 3:**
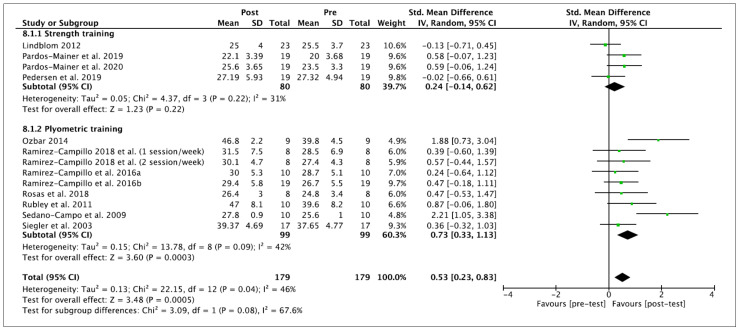
Forest plot of within-mode effect sizes with 95% confidence intervals (CIs) in vertical jump performance (cm). IV: inverse variance method; SD: standard deviation; Std: standardized.

Twelve studies were included in this systematic review and meta-analysis. Vertical jump height was measured in centimeters. The performance of training programs was associated with a moderate and significant increase in vertical jump performance (ES 0.53 (95% CI—0.11, 0.95), Z = 2.47 (*p* = 0.01)). There was a significant level of between-study heterogeneity (*I*^2^ = 69% (*p* = 0.0001)). Concerning the subgroup analyses, non-significant performance improvements were observed after ST (ES 0.24 (95% CI −0.14, 0.62), Z = 1.23 (*p* = 0.22)). A significant difference was observed for PT (ES 0.73 (95% CI—0.33, 1.13), Z = 3.48 (*p* = 0.0005)). No significant differences among subgroups were observed (*p* = 0.07). Within-mode ESs were small and moderate (ST: ES 0.24 (95% CI −0.14, 0.62), Z = 1.23 (*p* = 0.22); PT: ES 0.73 (95% CI—0.33, 1.13), Z = 3.60 (*p* = 0.0003)), respectively. No significant differences among subgroups were observed (*p* = 0.08). These results are displayed in Figure 2 (ST vs. PT) and Figure 3 (baseline vs. follow-up).

#### 3.4.2. Linear Sprint Time

**Figure 4:**
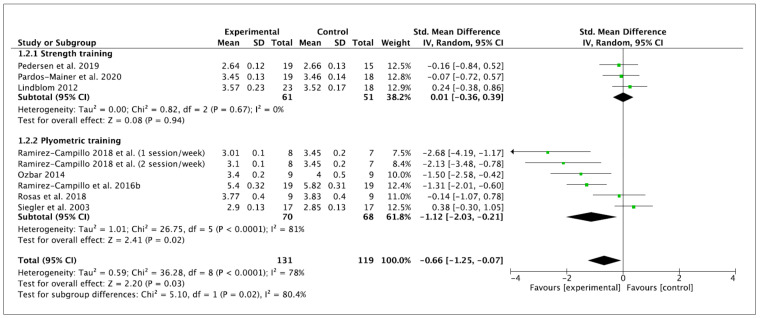
Forest plot of between-mode effect sizes with 95% confidence intervals (CIs) in time of linear sprint (s). IV: inverse variance method; SD: standard deviation; Std: standardized.

**Figure 5:**
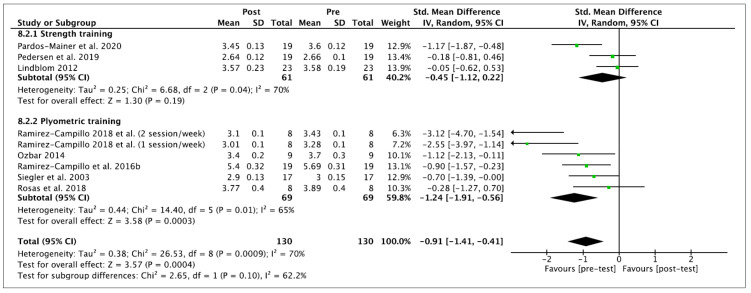
Forest plot of within-mode effect sizes with 95% confidence intervals (CIs) in time of linear sprint (s) e. IV: inverse variance method; SD: standard deviation; Std: standardized.

Nine effects were analyzed from 12 original studies. The linear sprint performance was measured in time (seconds). The performance of training programs was associated with a moderate and significant reduction in the time of linear sprint (ES −0.66 (95% CI −2.03, −0.21), Z = 2.20 (*p* = 0.03)). There was a significant level of between-study heterogeneity (*I*^2^ = 78% (*p* = < 0.0001)). Concerning the subgroup analyses, non-significant performance improvements were observed after ST (ES 0.01 (95% CI −0.36, 0.39), Z = 0.08 (*p* = 0.94)). A significant difference was observed for PT (ES −1.12 (95% CI −2.03, 0.21), Z = 2.41 (*p* = 0.02)). Significant differences among subgroups were observed (*p* = 0.02). Within-mode ESs were small and large (ST: ES −0.45 (95% CI −1.12, 0.22), Z = 1.30 (*p* = 0.19); PT: ES −1.24 (95% CI −1.91, 0.56), Z = 3.58 (*p* = 0.0003)), respectively. No significant differences among subgroups were observed (*p* = 0.10). These results are displayed in Figure 4 (ST vs. PT) and Figure 5 (baseline vs. follow-up).

#### 3.4.3. COD Time

**Figure 6:**
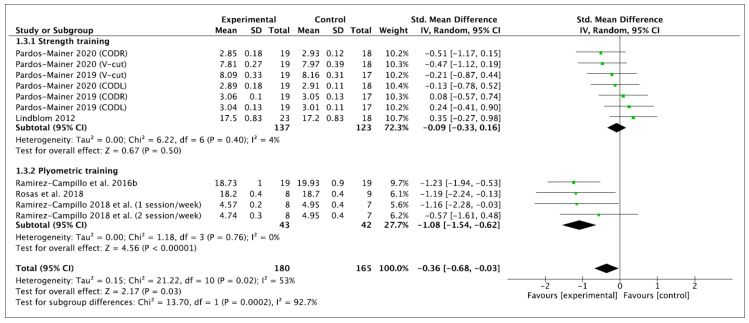
Forest plot of between-mode effect sizes with 95% confidence intervals (CIs) in the time of change of direction (s). IV: inverse variance method; SD: standard deviation; Std: standardized.

**Figure 7:**
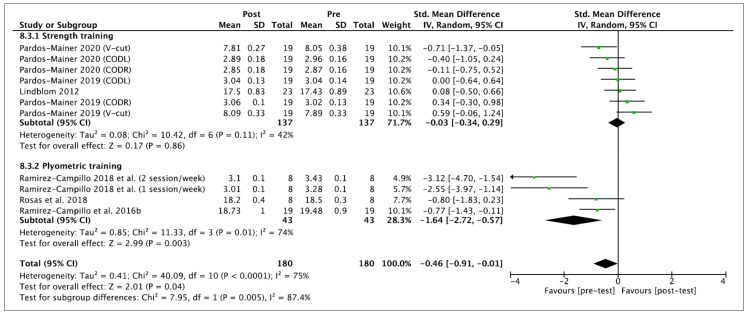
Forest plot of within-mode effect sizes with 95% confidence intervals (CIs) in the time of change of direction (s). IV: inverse variance method; SD: standard deviation; Std: standardized.

Seven effects were analyzed from 12 original studies. The COD performance was measured in time (seconds). The performance of training programs was associated with a small and significant reduction in the time of COD (ES −0.36 (95% CI −0.68, −0.03), Z = 2.17 (*p* = 0.03)). There was a significant level of between-study heterogeneity (*I*^2^ = 53% (*p* = 0.02)). Concerning the subgroup analyses, non-significant performance improvements were observed after ST (ES −0.09 (95% CI −0.33, 0.16), Z = 0.67 (*p* = 0.50)). A significant difference was observed for PT (ES −1.08 (95% CI −1.54, −0.62), Z = 2.17 (*p* = 0.03)). Significant differences among subgroups were observed (*p* = 0.0002). Within-mode ESs were small and large (ST: ES −0.03 (95% CI −0.34, 0.29), Z = 0.17 (*p* = 0.86); PT: ES −1.64 (95% CI −2.72, 0.57), Z = 2.99 (*p* = 0.003)), respectively. Significant differences among subgroups were observed (*p* = 0.005). These results are displayed in Figure 6 (ST vs. PT) and Figure 7 (baseline vs. follow-up).

### 3.5. Effect of Moderator Variables

A summary of the effect of moderator variables can be viewed in Table 4 and Table 5.

### 3.6. Strength Training

Subgroup analysis suggested high levels of between-group heterogeneity with session duration in linear sprint performance and total number of training session and session duration in COD performance, achieving statistical significance (*p* = 0.01).

Differences were trivial to small between each training type across subgroups in vertical jump and COD performance and trivial to large in linear sprint performance. In linear sprint performance, interventions with a total number of training sessions of less than 16 sessions produced moderate effects (ES −0.67 (95%CI = −1.64; 0.31), Z = 1.34 (*p* = 0.18)) compared to those that lasted longer than 16 sessions (ES −0.05 (95%CI = −0.62; 0.53), Z = 0.16 (*p* = 0.87)). Sessions that lasted longer than 30 min were substantially more effective (ES −1.17 (95%CI = −1.87; −0,48), Z = 3.31 (*p* = 0.0009)) than those that lasted less than 30 min (ES −0.05 (95%CI = −0.62; 0.53)), Z = 0.16 (*p* = 0.87)). In COD performance, interventions with a total number of training sessions of less than 16 sessions produced smaller effects (ES −0.40 (95%CI = −0.78; −0.03), Z = 2.12 (*p* = 0.03)) than those that lasted longer than 16 sessions (ES 0.24 (95%CI = −0.07; 0.55), Z = 1.51 (*p* = 0.13)). Sessions that lasted longer than 30 min were substantially more effective (ES −0.40 (95%CI = −0.78; 0.03), Z = 2.12 (*p* = 0.03)) than those that lasted less than 30 min (ES 0.24 (95%CI = −0.07; 0.55), Z = 1.51 (*p* = 0.13)). In vertical jump and COD performance, the level of heterogeneity was higher in subgroups with longer programs, greater training frequency, more training sessions and fewer minutes per session. In linear sprint performance, levels of heterogeneity were higher in subgroups with longer programs, greater training frequency and fewer training sessions.

### 3.7. Plyometric Training

Subgroup analysis suggested high levels of between-group heterogeneity, with program duration in COD performance achieving statistical significance (*p* < 0.001). Differences were small to large in vertical jump, trivial to very large in linear sprint and moderate to very large in COD performance. All subgroup variables in linear sprint and COD performance demonstrated a significant effect. In vertical jump performance, only interventions with a total number of training sessions of more than 16 sessions (ES −1.22 (95%CI = −0.60; 3.04), Z = 1.32 (*p* = 0.19)) and which lasted less than 30 min (ES 0.41 (95%CI = −0.07; 0.90), Z = 1.66 (*p* = 0.10)) did not demonstrate a significant effect. In vertical jump performance, the level of heterogeneity was higher in subgroups with shorter programs, lower training frequency and more training sessions and minutes per session. In linear sprint performance, levels of heterogeneity were higher in subgroups with shorter programs, greater training frequency, fewer training sessions and fewer minutes per session. The level of heterogeneity in COD performance was higher in subgroups with fewer training sessions.

## 4. Discussion

The main findings of this meta-analysis indicate that PT can be used instead of ST to target vertical jump, linear sprint and COD performance in female soccer players. This has important implications for coaches because it means that female soccer players can developed vertical jump, linear sprint and COD qualities and technical skills concurrently, thus representing a more performance-efficient approach to training.

### 4.1. Vertical Jump Performance

The within- and between-mode analyses reveal that PT provides better benefits than ST in enhancing vertical jump performance in female soccer players. The magnitude of the improvements was deemed trivial for ST (ES = 0.13) and moderate for PT (ES = 0.81). However, the differences observed among the within- and between-groups were not significant. Therefore, the present meta-analysis cannot provide conclusive information regarding the best program to increase vertical jump performance in female soccer players.

Several reviews and meta-analyses support the notion that PT is an effective training program for the improvement of vertical jump performance in female athletes [12,36,46]. On the contrary, to the authors’ knowledge, there have been no reviews conducted regarding the effects of ST on vertical jump performance in this population. The main reason is that less research is available for this population and, therefore, more studies are needed.

The purpose of ST is to promote maximal strength and muscular hypertrophy to improve physical performance [22], and this method has often been used by physical trainers in soccer training routines [7,47]. Two studies by Pardos-Mainer et al. [5,6] found that ST exerted a borderline small–moderate effect on vertical jump performance whilst Lindblom et al. [39] and Pedersen et al. [38] resulted only a trivial ES, and even the effect was negative. It is possible that the exercises included in the ST programs do not demonstrate a significant transference effect to soccer-specific physical performance and conditioning programs with higher load and intensity would be necessary in order to benefit from the training [8,35]

Moreover, if we observe the different exercises used in ST of the current meta-analysis, it can be argued that there is low resemblance between the exercises carried out and the evaluated CMJ performance test. These exercises were generally carried out at slow speeds, while the CMJ test included high-speed components.

PT concerns exercises that have the aim to improve muscle, mainly through the use of jump training [48,49]. Plyometric exercises represent a natural part of majority sport movement because they involve jumping, hopping and skipping [46,47,50]. Ozbar et al. [42] and Sedano-Campo et al. [43] found that PT exerted a large effect and Rubley et al. [44] found a moderate effect on vertical jump performance, whilst the rest of the PT studies [36,37,40,41] resulted only in a small ES. These results are in line with the results of two meta-analyses which showed that PT increases vertical jump performance for female athletes [8,35].

The aforementioned magnitude differences in vertical jump performance after ST and PT among female soccer players may be due to the diversity of training programs (e.g., frequency, duration, total time and total number of ST and PT sessions). To analyze this possibility, the effects of potential moderator variables were explored.

Subgroup analyses of programming parameters revealed that ST interventions were more effective with longer study durations (8 weeks or more), greater training frequency (2 sessions or more per week), more training sessions (16 or more) and longer session times (30 min or more) to improve vertical jump performance. However, only four studies [5,6,38,39] provided data and, owing to the homogeneity of programming parameters used across studies, more research, utilizing varying study durations, amounts of sessions, training sessions and session times, should be carried out to establish more robust recommendations regarding these parameters.

On the other hand, certain programming characteristics of PT interventions, such as longer study durations (8 weeks or more), reduced training frequency (less than 2 sessions per week), more training sessions (16 or more) and longer session times (30 min or more), could enhance the effectiveness of vertical jump performance, although there is no suggestion that these factors are necessarily synergistic when combined. Regarding PT frequency, interventions with less than two sessions per week [40,42,44] produced a moderate effect (ES: 1.00), while those with two or more sessions per week [36,37,40,41,45] also produced a moderate but weaker effect (ES: 0.62). Then, such ES values must be interpreted cautiously. In this sense, the large effect observed in training sessions and session times may be inflated, probably related with the results from Ozbar et al. [42], Sedano-Campo et al. [43] and Rubley et al. [44]. Two [38,40] of these three studies showed a moderate–large effect on vertical jump performance (ES = 0.87–2.21) after the PT program. Such substantial improvements may be related to the initial vertical jump values of female soccer players, which are too high compared to the rest of the studies included in the current meta-analysis. Furthermore, Sedano-Campo et al. [43] performed the PT intervention with elite female soccer players, with an average of 10 h of training a week. Then, the characteristics of participants may explain the moderate effect (ES = 0.87) increase in vertical jump performance. Finally, no significant subgroup differences were noted for any of the moderator variables.

In general, the evidence suggests that the moderator roles of ST in vertical jump performance in female soccer player are not clear and more research is necessary. Meanwhile, the moderator roles of PT in vertical jump performance are more conclusive and such information may aid sports coaches and trainers in selecting programming characteristics of PT in this population.

### 4.2. Liner Sprint Time

In the present meta-analysis, the within-mode analyses reveal that PT shows better benefits in enhancing the time of linear sprint in female soccer players than ST. In addition, the improvements were significant for the PT program. The magnitude of the improvements was deemed trivial for ST (ES = 0.01) and large for PT (ES = −1.12). The subgroup differences were significant. However, these results must be interpreted conservatively. Between-mode analysis provided a greater effect in both types of programs. ST produced a small effect (ES = −0.45) while PT produced a large effect (ES = −1.24). Moreover, no significant subgroup differences were noted for linear sprint performance (*p* = 0.10). It is interesting to observe the between-mode analysis due to the comparison between the same ST or PT group (intra-group), whilst within-mode analysis is a comparison between EG and CG of ST or PT training and, occasionally, this mode of analysis does not represent the reality of the results. In this sense, Pardos-Mainer et al. [5] demonstrated a large effect (ES = −1.17) in linear sprint performance after an ST which is combined with power exercises. Similar results have been found by Ozbar et al. [42] (ES = −1.12) after a PT. It is well acknowledged that horizontal force production has an important application in sprint acceleration performance [51]. Both PT and ST incorporated horizontal stimulus, and this may have increased the chances of gaining adaptations. Hence, these results highlight the importance of developing both lower body strength and power, which may enhance linear sprint performance in female soccer players.

Based on the data presented in Table 4 and Table 5, on the one hand, certain ST programming characteristics, such as longer study durations (8 weeks or more), greater training frequencies (2 sessions or more per week), fewer training sessions (16 or less) and longer session times (30 min or more), could enhance the effectiveness of linear sprint performance. Indeed, significant subgroup differences were noticed (*p* = 0.01) regarding the session time. Regarding the duration and total number of ST sessions, interventions with durations of 30 min or more per session and 16 or fewer sessions demonstrated large (ES = −1.17) and moderate (ES = −0.67) effects, respectively. However, these findings are not clear but this could be due to the relatively low number of studies in this field [36,37], thus necessitating more research to clarify the time course of adaptation to ST in linear sprint performance in female soccer players. On the other hand, PT program characteristics such as short study durations (less than 8 weeks), lower training frequencies (2 sessions per week or less), fewer training sessions (16 or less) and short session times (less than 30 min) could improve the effectiveness of linear sprint performance. Nevertheless, no significant subgroup differences were noted for moderator variables. Characteristics of PT programs with low dosage may maximize one’s probability of improving linear sprint performance and other meta-analysis studies support this finding [49].

It can be concluded that a PT program can enhance liner sprint performance over a distance down to 30 m in length. In addition, PT results in an increase in linear sprint performance, especially over the initial meter, between 15 and 30 m distances. A meaningful portion of the PT exercises in studies included in the current meta-analysis implicated slow stretch shortening cycle (SSC) muscle actions. These actions mimic those encountered during the acceleration phase of a sprint [52,53] compared to the faster SSC muscle actions of the maximal velocity of a sprint [52]. For this reason, the specificity principle of training may help to explain the enhancement in the linear sprint after a PT program [54]. In this sense, coaches and trainers should consider incorporating sprint specific exercises as part of the PT program.

### 4.3. COD Time

Within- and between-mode analyses reveal that PT is more beneficial in improving the time of COD performance in female soccer players than ST. The magnitude of the enhancements was deemed trivial for ST (ES = −0.03) and large for PT (ES = −1.64). Moreover, the subgroup differences were significant. Neuromuscular adaptations during the initial weeks in ST and PT are important [55,56]. Neural adaptations and improvement of motor unit recruitment are mechanisms that can lead to an enhancement in COD performance [56]. Improvements in COD ability require rapid force development, the eccentric strength of the thigh muscles and a rapid switch from eccentric to concentric muscle action in the leg-extensor muscles, and it seems that PT can improve these factors [57,58]. It is probable that PT studies [36,37,40] showed very large to moderate effects (ES = −3.12 to −0.77) because these programs incorporated vertical, horizontal and unilateral jumps that increased COD performance. However, Pardos-Mainer et al. [5] observed a moderate effect (ES = −0.71) after ST combined with isometric exercises. The isometric strength seems to be decisive to optimize the triple extension during COD tests, as a result of permitting the correct alignment of the lower limbs to then subsequently reaccelerate thereafter [59].

Further subgroup analyses of programming parameters also revealed some interesting findings. ST interventions were more effective with longer study durations (8 weeks or more), greater training frequencies (2 sessions or more per week), fewer training sessions (16 or less) and longer session times (30 min or more). Meanwhile, PT interventions were more effective with longer study durations (8 weeks or more), lower training frequencies (less than 2 sessions per week), fewer training sessions (16 or less) and longer session times (30 min or more). However, these results must be interpreted cautiously being as there are no existing ST and PT studies which examine these programming parameters.

On the one hand, significant ST subgroup differences were noted for total number and duration of sessions (*p* = 0.01); nevertheless, two studies [4,6] reported more than one outcome to evaluate the COD performance and may have overestimated the precision of this ability. On the other hand, significant subgroup differences were noticed regarding the PT duration (*p* = <0.01). However, ES values must be interpreted conservatively. The very large effect (ES = −2.80) observed with programs which were 8 weeks or longer may be inflated, probably related to the results from one [36] of the three studies that observed duration of training.

Overall, the evidence suggests that PT significantly improves COD performance; nevertheless, we cannot strongly recommend optimal training variables to improve COD performance in female soccer players. Researchers are therefore encouraged to conduct studies examining different ST and PT programming parameters in female soccer players.

### 4.4. Limitations

Besides the inherent limitations associated with the meta-analytic technique itself, a number of specific limitations of the current meta-analysis have to be considered. This meta-analysis does not allow coaches and trainers to provide definite ST or PT programs to enhance vertical jump, linear sprint and COD performance because mainly no significant subgroup differences were noticed according to moderator variables. However, the current meta-analysis indicates that PT improves, to a greater extent, these variables of performance than ST; nevertheless, the current results should be interpreted with caution and confirmed in the future. A limitation of the present body of literature is the relatively high number of researchers who did not incorporate a control group into their study design. Six studies [7,60,61,62,63,64] were excluded from the current meta-analysis because they did not provide any, or sufficient, control group data. The recruitment of individuals to studies can be difficult and the addition of a control group is not always possible in female soccer due to the smaller number of female players in comparison to their male counterparts. In this regard, we encourage future studies to compare the effects of ST and PT against a control group to elucidate which are the most beneficial in vertical jump, linear sprint or COD performance in female soccer players. Furthermore, due to the lack of studies in this population, we decided to pool the data of youth and adult female players in the meta-analysis to include a broader number of studies. However, the specific information of each study, presented in Table 2 and Table 3, together the within- and between- values provided in Figure 2, Figure 3, Figure 4, Figure 5, Figure 6 and Figure 7, allows interested readers to re-conduct the meta-analysis if they wish to delimitate the range of maturity status and age of studies further.

## 5. Conclusions

The findings of this systematic review and meta-analysis suggest that PT seems to provide better benefits than ST to improve vertical jump, linear sprint and COD performance in female soccer players. However, significant limitations in the current literature prevent assured PT and ST prescriptions recommendations being made. Based on our results, it seems that theses physical performance gains may be optimized by the use of vertical, horizontal and unilateral jumps at high speed and these exercises represent a natural part of the majority of sport movement because they involve jumping, hopping and skipping. In addition, exercises included in ST were generally carried out at slow speeds and could decrease the performance. Further research is needed in adolescent, recreational, elite and adult female soccer players to investigate the effects of PT and ST on performance. Furthermore, longer-term studies are also needed to determine and compare the long-term effectiveness of both training programs on performance.

## Figures and Tables

**Table 1:** The Physiotherapy Evidence Database (PEDro) scale ratings.

Studies	N°1	N°2	N°3	N°4	N°5	N°6	N°7	N°8	N°9	N°10	N°11	Total ^1^
Lindblom et al., 2012 [39]	1	1	1	1	1	0	0	0	0	1	1	7
Ozbar et al., 2014 [42]	0	1	0	1	0	0	0	1	0	1	1	5
Pardos-Mainer et al., 2019 [5]	0	1	1	1	1	0	0	1	0	1	1	7
Pardos-Mainer et al., 2020 [6]	0	1	1	1	1	0	0	1	0	1	1	7
Pedersen et al., 2019 [38]	1	1	1	1	1	0	0	1	0	1	1	8
Ramirez-Campillo 2016 b [41]	0	1	1	1	1	1	1	1	0	1	1	7
Ramirez-Campillo 2016 a [36]	0	1	1	1	1	0	0	1	0	1	1	9
Ramirez-Campillo 2018 (1 session/wk.) [40]	1	1	1	1	0	0	1	0	0	1	1	7
Ramirez-Campillo 2018 (2 session/wk.) [40]	1	1	1	1	0	0	1	0	0	1	1	7
Rosas et al., 2018 [37]	0	1	1	1	1	1	1	1	0	1	1	9
Rubley et al., 2011 [44]	0	0	0	1	0	0	0	1	0	1	1	4
Sedano del Campo et al., 2009 [43]	0	1	0	1	0	0	0	1	0	1	1	5
Siegler et al., 2003 [45]	0	0	0	1	0	0	0	1	0	1	1	4

^1^ The total number of points from a possible maximal of 10.

**Table 2:** Characteristics of study participants of strength training.

Study	Study Group	N	Age (Years)	BM (kg)	Height (cm)	SST	Wks	F	T	D	Exercise Type	Test	Response
Lindblom et al.	ST (FIFA 11+)	23	14.2 ± 0.7	53.9 ± 8.6	165 ± 6.5	Yes	11	2	22	15	One-legged knee squat, pelvic lift, two-legged knee squat, the bench, the lunge and jump/landing	CMJ 20-m linear sprint Illinois agility test	=CMJ =20-m linear sprint =Illinois agility test
	Control	18	14.2 ± 1.1	51.6 ± 7.4	164.2 ± 6.1								
Pardos-Mainer et al.	ST (FIFA 11+)	19	12.5 ± 0.4	51.2 ± 7.7	153.7 ± 6.9	Yes	10	2	20	20	Running, lower extremities’ strength, balance, plyometric, agility and COD exercises	CMJ V-cut test	↑ CMJ ↓ V-cut test
	Control	17	13.1 ± 0.3	55.9 ± 8.2	160.8 ± 4.9								
Pardos-Mainer et al.	ST (CSPT)	19	16.2 ± 0.9	55.9 ± 5.5	159.8 ± 5.4	Yes	8	2	16	35	The diver, one-legged pelvic tilt, single leg box step-up, forward lunge, backward lunge, one-legged hip thrust, eccentric box drops, Russian belt posterior chain, Russian belt anterior chain, plank, lateral plank and lumbar bridge	CMJ 20-m linear sprint V-cut test	↑ CMJ ↑ 20-m linear sprint ↑ V-cut test
	Control	18	15.6 ± 0.9	54.1 ± 8.8	159.7 ± 4.9								
Pedersen et al.	ST	18	18 ± 3	62 ± 6	167 ± 6	Yes	5	2	10	NR	90°squat with load and Nordic hamstring exercises	CMJ 15-m linear sprint	=CMJ =15-m linear sprint
	Control	15	19 ± 2	63 ± 10	168 ± 5								

Note: BM: Body mass; CMJ; Countermovement jump; CSPT: Combined strength and power training; F: Frequency (per wk.); T: Total sessions; D: Mean session duration (min); NR: Non-reported; ST: Strength training; SST: Indicates if the participants had previous systematic experience with ST; FIFA: Federation international football association; COD: Change of direction.

**Table 3:** Characteristics of study participants of plyometric training.

Study	Study Group	N	Age (years)	BM (kg)	Height (cm)	SPT	Wks	F	T	D	Exercise Type	Test	Response
Ozbar et al.	PT	9	18.3 ± 2.6	58.8 ±7.8	163.1 ± 5.3	Yes	1	8	8	30–40	Variety of plyometric exercises designed for the lower extremity (i.e., bilateral and unilateral DJs, CMJs and SLJ)	CMJ 20-m linear sprint	↑ CMJ ↑ 20-m linear sprint
	Control	9	18 ± 2	54.4 ± 6.1	159.4 ± 5.1								
Ramirez-Campillo 2016 a	PT	10	22.9 ± 2.1	56.8 ± 5.4	164 ± 9	No	2	6	12	NR		CMJ	↑ CMJ
	Control	10	22.5 ± 2.1	60.1 ± 7.5	161± 6								
Ramirez-Campillo 2016 b	PT	19	22.4 ±2.4	60.7 ± 9.3	161 ± 5	No	2	6	12	40		CMJ 30-m linear sprint COD speed test	↑ CMJ ↑ 30-m linear sprint ↑ COD speed test
	Control	19	20.5 ± 2.5	60.2 ± 9.3	159 ± 6								
Ramirez-Campillo 2018 (1 session/wk.)	PT	8	22.8 ± 4.3	54.9 ± 3.7	158 ± 3	No	1	8	8	6–20		CMJ 15-m linear sprint COD speed test	↑ CMJ ↑ 15-m linear sprint ↑ COD speed test
	Control	7	20.1 ± 1.8	55.3 ± 3.3	160.1 ± 5								
Ramirez-Campillo 2018 (2 session/wk.)	PT	8	21.4 ± 2.5	59.6 ± 8.5	157.6 ± 4.8	No	2	8	16	6–20		CMJ 15-m linear sprint COD speed test	↑ CMJ ↑ 15-m linear sprint ↑ COD speed test
	Control	7	20.1 ± 1.8	55.3 ± 3.3	160.1 ± 5								
Rosas et al.	PT	8	22.8 ± 2.1	61.1 ± 8.3	164 ± 8	No	2	6	12	NR		CMJ	↑ CMJ
	Control	9	24 ± 2.7	58.5 ± 7.2	132 ± 4								
Rubley et al.	PT	10	13.4 ± 0.5	50.8 ± 5.1	162.5 ± 5.6	No	1	12	12	NR		CMJA	↑ CMJA
	Control	6	NR	NR	NR								
Sedano- Campo et al.	PT	10	22.8 ± 2.1	58.5 ± 9.3	163 ± 7	Yes	3	12	36	46–60		CMJ	↑ CMJ
	Control	10	23 ± 3.2	56.9 ± 7.4	161.5 ± 5.4								
Siegler et al.	PT	17	16.5 ± 0.91	61.4 ± 9.43	167.4 ± 4.6	No	2 (1–3)	10	20	10–15		CMJA 20-m linear sprint	↑ CMJA ↑ 20-m linear sprint
	Control	17	16.2 ± 1.4	58 ± 7.23	166.7 ± 4.7								

BM: Body mass; DJ: Drop jump; CMJ; Countermovement jump; CMJA: CMJ with arm swing; NR: Non-reported; PT: Plyometric training; SPT: Indicates if the participants had previous systematic experience with PT; SLJ: Standing long jump.

**Table 4:** Effect of moderator variables with 95% confidence intervals in strength training.

Variable	Subgroup	Effect Size with 95% Confidence Interval	Effect Descriptor	Groups	*n*	Within-Group I^2^ (%)	Within-Group *p* ^a^	Between-Group I^2^ (%)	Between-Group *p* 1 ^b^
CMJ	<8 weeks	−0.02 (−0.66; 0.61)	Trivial	1	61	NE	0.94	0.0	0.39
	≥8 weeks	0.33 (−0.16; 0.81)	Small	3	19	44.0	0.18		
	<2 sessions/week	NE							
	≥2 sessions/week	0.24 (−0.14; 0.62)	Small	4	80	31.0	0.22		
	≤16 sessions	0.28 (−0.32; 0.88)	Small	2	38	43.0	0.36	0.0	0.88
	>16 sessions	0.21 (−0.48; 0.90)	Small	2	42	61.0	0.55		
	<30 min/session	0.21 (−0.48; 0.90)	Small	2	42	61.0	0.55	44.0	0.18
	≥30 min/session	0.59 (−0.06; 1.24)	Small	1	19	NE	0.08		
Sprint test	<8 weeks	−0.18 (−0.81; 0.46)	Trivial	1	19	NE	0.59	0.0	0.43
	≥8 weeks	−0.59 (−1.70; 0.51)	Small	2	42	83.0	0.29		
	<2 sessions/week	NE							
	≥2 sessions/week	−0.45 (−1.12; 0.22)	Small	3	61	70.0	0.19		
	≤16 sessions	−0.67 (−1.64; 0.31)	Moderate	2	38	0.77	0.18	12.5	0.28
	>16 sessions	−0.05 (−0.62; 0.53)	Trivial	1	23	NE	0.87		
	<30 min/session	−0.05 (−0.62; 0.53)	Trivial	1	23	NE	0.87	83.3	0.01
	≥30 min/session	−1.17 (−1.87; −0.48)	Large	1	19	NE	<0.001		
COD tests	<8 weeks	NE							
	≥8 weeks	−0.03 (−0.34; 0.29)	Trivial	7	137	42.0	0.86		
	<2 sessions/week	NE							
	≥2 sessions/week	−0.03 (−0.34; 0.29)	Trivial	7	137	42.0	0.86		
	≤16 sessions	−0.40 (−0.78; −0.03)	Small	3	57	0.0	0.03	85.1	0.01
	>16 sessions	0.24 (−0.07; 0.55)	Trivial	4	80	0.0	0.13		
	<30 min/session	0.24 (−0.07; 0.55)	Trivial	4	80	0.0	0.13	85.1	0.01
	≥30 min/session	0.40 (−0.18; −0.03)	Small	3	57	0.0	0.03		

^a^: Test of null (2-tail), mixed model; ^b^: *p* value, heterogeneity, total between, mixed model; NE: Not estimable.

**Table 5:** Effect of moderator variables with 95% confidence intervals in plyometric training.

	Subgroup	Effect Size with 95% Confidence Interval	Effect Descriptor	Groups	*n*	Within-Group I^2^ (%)	Within-Group *p* ^a^	Between-Group I^2^ (%)	Between-Group *p* ^b^
CMJ	<8 weeks	0.41 (−0.06; 0.87)	Small	3	37	0.0	0.08	52.2	0.15
	≥8 weeks	0.96 (0.37; 1.56)	Moderate	6	62	57.0	0.002		
	<2 sessions/week	1.00 (0.19; 1.80)	Moderate	3	27	46.0	0.01	0.0	0.42
	≥2 sessions/week	0.62 (0.15; 1.08)	Moderate	6	72	43.0	0.009		
	≤16 sessions	0.61 (0.27; 0.95)	Moderate	7	72	1.0	0.0004	0.0	0.52
	>16 sessions	1.22 (−0.60; 3.04)	Large	2	27	86.0	0.19		
	<30 min/session	0.41 (−0.07; 0.90)	Small	3	33	0.0	0.10	58.8	0.12
	≥30 min/session	1.44 (0.25; 2.63)	Large	3	38	78.0	0.02		
Sprint test	<8 weeks	−1.39 (−2.29; −0.48)	Large	5	50	72.0	0.003	0.0	0.40
	≥8 weeks	−0.90 (−1.57; −0.23)	Moderate	1	19	NE	0.008		
	<2 sessions/week	−1.75 (−3.14; −0.36)	Large	2	17	62.0	0.01	0.0	0.38
	≥2 sessions/week	−1.03 (−1.82; −0.23)	Moderate	4	52	68	0.01		
	≤16 sessions	−1.42 (−2.29; −0.56)	Large	5	52	80	0.02	39.2	0.20
	>16 sessions	−0.70 (−1.39; 0)	Small	1	17	NE	0.05		
	<30 min/session	−2.01 (−3.64; −0.37)	Very Large	3	33	82.0	0.02	0	0.37
	≥30 min/session	−1.12 (−2.13; −0.11)	Large	1	9	NE	0.03		
COD test	<8 weeks	−0.78 (−1.34; −0.22)	Moderate	2	27	0.0	0.006	91.0	0.0009
	≥8 weeks	−2.80 (−3.86; −1.75)	Very Large	2	16	0.0	<0.00001		
	<2 sessions/week	−1.16 (−2.28; −0.03)	Large	1	8	NE	0.04	0.0	0.77
	≥2 sessions/week	−0.97 (−1.48; −0.47)	Moderate	3	35	0.0	0.0002		
	≤16 sessions	−1.64 (−2.72; −0.57)	Large	4	43	74.0	0.003	NE	NE
	>16 sessions	NE							
	<30 min/session	−2.80 (−3.86; −1.75)	Very large	2	16	0.0	<0.00001	NE	NE
	≥30 min/session	NE							

^a^: Test of null (2-tail), mixed model; ^b^: *p* value, heterogeneity, total between, mixed model; NE: Not estimable.

## Data Availability

The datasets generated and analyzed for this study can be requested by correspondence authors in epardos@usj.es and dlozano@usj.es.
